# Acute phase clinical manifestations of patients with Vogt-Koyanagi-Harada disease in Southern China

**DOI:** 10.1186/s12886-023-02952-y

**Published:** 2023-05-05

**Authors:** Chuhua Zhang, Yun Wang, Yun Chen, Hui Zhou, Qiuxia Hong, Xiuying Yu, Tsz Kin Ng, Ling-Ping Cen

**Affiliations:** 1 Joint Shantou International Eye Center of Shantou University and The Chinese University of Hong Kong, North Dongxia Road, Shantou, Guangdong China; 2 Shantou University Medical College, Shantou, 515041 Guangdong China; 3 Department of Ophthalmology and Visual Sciences, The Chinese University of Hong Kong, Hong Kong Special Administrative Region, China

**Keywords:** Vogt-Koyanagi Harada disease, Acute phase, Clinical manifestations, Corticosteroid therapy, Visual outcome

## Abstract

**Backgrounds:**

To characterize the acute phase clinical manifestations and visual outcomes of the patients with Vogt-Koyanagi Harada (VKH) disease in southern China.

**Methods:**

In total, 186 patients with acute-onset VKH disease were recruited. The demographic data, clinical signs, ophthalmic examinations, and visual outcomes were analyzed.

**Results:**

Among the 186 VKH patients, 3 were diagnosed as complete VKH, 125 as incomplete VKH, and 58 as probable VKH. All patients visited the hospital within 3 months of onset and complained of decreased vision. For the extraocular manifestations, 121 patients (65%) referred neurological symptoms. Anterior chamber activity was negative in most eyes within an onset of 7 days, which increased slightly with onset beyond 1 week. Exudative retinal detachment (366 eyes, 98%) and optic disc hyperaemia (314 eyes, 84%) were commonly observed at presentation. A typical ancillary examination helped with the diagnosis of VKH. Systemic corticosteroid therapy was prescribed. The logMAR best-corrected visual acuity improved significantly from 0.74 ± 0.54 at baseline to 0.12 ± 0.24 at the 1-year follow-up visit. The recurrence rate was 18% in the follow-up visits. Erythrocyte sedimentation rate and C-reactive protein were significantly correlated to VKH recurrences.

**Conclusion:**

Posterior uveitis, followed by mild anterior uveitis, is the typical initial manifestation in the acute phase of Chinese VKH patients. Visual outcome improvement is promising in most patients receiving systemic corticosteroid therapy in the acute phase. Detection of the clinical features at the initial onset of VKH could facilitate early treatment and better vision improvement.

**Supplementary Information:**

The online version contains supplementary material available at 10.1186/s12886-023-02952-y.

## Background

The Vogt-Koyanagi-Harada (VKH) disease is a rare autoimmune inflammatory disorder of unknown origin that affects the melanin-containing tissues, including the meninges, ear, skin, and uvea [1]. The VKH incidences vary depending on the geographic locations and ethnicities. It is more common in Asian, Middle Eastern, Hispanic, and Native American populations [1]. In China, it accounts for 15.9% of panuveitis [2]. The clinical features of the VKH disease are significantly different in various ethnics and study groups [3–8].

The VKH disease shares similar clinical manifestations with various ocular and neurological diseases. The spectrum of misdiagnosis includes optic neuritis, retinal detachment, anterior granulomatous uveitis, migraine, and intracranial hypertension [9]. The clinical presentations of VKH disease can mimic inflammatory diseases, sympathetic ophthalmia, and acute angle closure [10–12]. Around 14% of patients with the VKH disease could be initially misdiagnosed as central serous chorioretinopathy [13]. It has been shown that 9% of the patients with VKH disease are incorrectly diagnosed at the initial onset [9]. Critically, the window of the therapeutic opportunity has been predicted within 2—3 weeks after the initial disease onset [14]. Timely and adequate treatment benefits vision improvement and avoids chronic evolution 14. A delay in diagnosis could lead to a delay in treatment and poor visual outcome. Therefore, it is crucial to recognize the early clinical presentations of the VKH disease.

Previous studies have reported the clinical spectrums of the VKH disease at various stages in Chinese patients; yet, the clinical manifestations of the patients with the VKH disease in the acute phase should be explored despite the relatively high prevalence rate of the VKH disease in China [7, 11, 15]. Herein, in this study, we aimed to characterize the longitudinal profile of the patients with new-onset VKH disease referred to our eye hospital. We evaluated the visual outcome after systemic corticosteroid therapy in the acute phase.

## Methods

### Study subjects

This is a retrospective study on the inpatients diagnosed with VKH disease at the Joint Shantou International Eye Center of Shantou University and the Chinese University of Hong Kong from 2013 to 2019. All enrolled patients met the revised diagnostic criteria for the VKH disease [16]: 1) No history of penetrating ocular trauma; 2) No evidence of other ocular or systemic diseases; 3) Manifestation of bilateral ocular disease: either early diffuse choroiditis, equivocal fundus findings with evidence of choroiditis by fluorescein angiography and diffuse choroidal thickening by ultrasonography, or late manifestation of disease, including a history of the conditions mentioned above, ocular depigmentation (sunset glow fundus or Sugiura sign), and other ocular signs (nummular chorioretinal depigmentation, retinal pigment epithelium clumping or migration or recurrent/chronic anterior uveitis); 4) Neurological/auditory findings: meningismus, tinnitus or cerebrospinal fluid pleocytosis; 5) Integumentary finding: alopecia, poliosis or vitiligo. The patients diagnosed with complete VKH must meet point 1 to 5; incomplete VKH for point 1 to 3 and either 4 or 5; and probable VKH disease as the isolated ocular disease and meeting point 1 to 3. The patients within 3 months of onset were in the acute stage. Systemic diseases that might mimic the VKH disease, such as lupus, syphilis, tuberculosis, and Lyme disease, were ruled out according to systemic symptoms and related routine laboratory tests. The patients' demographics, ocular and extraocular manifestations, ancillary examination results, treatment modalities, and visual outcomes were retrieved from the electronic medical records. The study protocol was approved by the Ethics Committee for Human Medical Research at the Joint Shantou International Eye Center of Shantou University and the Chinese University of Hong Kong, which is in accordance with the Tenets of the Declaration of Helsinki. The Ethics Committee has approved the exemption of writing consent from patients as it was a retrospective study. Anonymisation of any personal information was sufficiently guaranteed throughout the whole process of the study.

### Ocular examinations and blood tests

All study subjects received complete ocular examinations, including best-corrected visual acuity (BCVA) using the Snellen chart, intraocular pressure (IOP), and slit-lamp microscopy. Anterior segment inflammatory cells were graded using the Standardized Uveitis Nomenclature guideline [17]. The BCVA was presented as the logarithm of the minimum angle of resolution (logMAR) values. The examination results of fundus fluorescein angiography (FFA), optical coherence tomography (OCT), B-scan ultrasonography, and ultrasound biomicroscopy (UBM) at the initial visit before the treatment were also collected. The patients routinely received the X-ray examination and infectious serology tests, including syphilis, upon admission. The outcomes of routine blood tests, such as complete blood count (CBC), hepatorenal function test, erythrocyte sedimentation rate (ESR), C-reactive protein, anti-streptolysin O titer (ASO), rheumatoid factor, human leukocyte antigen B27 (HLA-B27) tests, antinuclear antibody (ANA) tests, and extractable nuclear antigen (ENA) panel, were retrieved. The type and duration of corticosteroid therapy and the follow-up visits were also retrieved.

### Corticosteroid treatment

After the admission, ocular examinations, and blood tests, the VKH patients were initially administered with oral corticosteroids (prednisone at 1 – 1.5 mg/kg body weight per day) or intravenous dexamethasone (10 mg per day) in severe cases. Topical corticosteroids and cycloplegic drops were also administered to manage the disease. After discharge from the hospital, the patients were asked to continue the oral corticosteroid therapy. The adjustment or cessation of the corticosteroid therapy was advised for the patients in their follow-up visits. Recurrence was documented according to the symptoms and signs of inflammation, and the corresponding OCT findings during their regular follow-up visits for minimum 9 months.

### Disease onset categorization

The patients were divided into 5 groups according to the interval between the onset of the symptoms and the first admission to our hospital. Group 1 consisted of the patients consulted within 4 days after the uveitis attack; Group 2 of the patients admitted to our hospital between 5—7 days; Group 3 of the patients with the interval between 1—2 weeks; Group 4 of those with the interval between 2 weeks and 1 month; and Group 5 of those admitted to our hospital between 1—3 months.

### Statistical analysis

The parametric data was analyzed by the independent T-test or one-way analysis of variance (ANOVA) test, while the non-parametric data was analyzed by the Mann–Whitney U test or Kruskal–Wallis H test. The χ^2^ test or Fisher's exact test was used for the comparisons of the categorical data. Post-hoc Bonferroni test was used for multiple testing corrections. Paired sample T test was performed to compare the differences of the dynamic changes. All statistical analyses were performed using a commercially available software (IBM SPSS Statistics 23; SPSS Inc., Chicago, IL). *P* < 0.05 was considered statistically significant.

## Results

### Demographics of the study subjects

Ninety-seven (52%) of the 186 enrolled study subjects were male, and 89 (48%) were female. The overall mean age at the disease onset was 39.68 ± 13.34 years (range 9 – 72 years) (Supplementary Fig. 1). All patients were ethnic Han Chinese. Three patients were classified as complete VKH, 125 as incomplete VKH, and 58 as probable VKH according to the revised diagnostic criteria for the VKH disease from International Study Committee [16]. The majority of the patients (184, 99%) were bilaterally involved according to the chief complaint at the initial onset. The 2 patients with unilateral complaint onset were diagnosed with the VKH disease as the FFA revealed bilateral delayed choroidal perfusion, subretinal pooling, and optic nerve staining, whereas the B-scan revealed bilateral choroidal thickening. For the systemic diseases, 14 patients revealed a history of hypertension, and 5 patients with diabetes. The patients were divided into 5 groups according to the interval between the symptoms and first admission. Forty (21%) patients met the criteria of Group 1, 53 (28%) for Group 2, 60 (32%) for Group 3, 29 (16%) for Group 4, and 4 (3%) for Group 5. The onset ages were significantly different between Group 2 and Group 3 (adjusted *p* = 0.006). Table 1 presents the detailed demographics and clinical characteristics at the first visit.

**Table 1:** Demographics and clinical characteristics at the first visit

Variables	Total	GROUP 1	GROUP 2	GROUP 3	GROUP 4	GROUP 5	*p-value*
Numbers, *n* (%)	186	40 (21)	53 (28)	60 (32)	29 (16)	4 (3)	
Mean age (years)	39.68 ± 13.34	37.3 ± 13.03	35.45 ± 11.69	44 ± 13.48	40.24 ± 13.31	50.75 ± 15.65	**0.003**^a^
Gender, *n* (%)							0.437^b^
Male	97 (52)	18 (45)	32 (60)	28 (47)	16 (55)	3 (75)	
Female	89 (48)	22 (55)	21 (40)	32 (53)	13 (45)	1 (25)	
Onset-admission interval (days)	10.77 ± 9.2	3.05 ± 0.88	6.26 ± 0.88	11.32 ± 1.97	24.9 ± 5.69	37.25 ± 28.11	
Duration of corticosteroid treatment (days)	7.85 ± 3.33	7.9 ± 3.36	7.99 ± 3.35	7.88 ± 3.35	7.99 ± 3.37	8.25 ± 3.06	
Bilateral onset, *n* (%)	184 (99)	39 (98)	53 (100)	59 (98)	29 (100)	4 (100)	0.815^b^
Clinical types, *n* (%)							0.073^b^
Complete	3 (2)	0	2 (4)	0	1 (3)	0	
Incomplete	125 (67)	24 (60)	42 (79)	37(62)	20 (69)	2 (50)	
Probable	58 (31)	16 (40)	9 (17)	23(38)	8 (28)	2 (50)	
Systemic disease, *n*							
Hypertension	14	2	3	6	3	0	0.783^b^
Diabetes	5	1	1	1	2	0	0.698^b^
Chief complaint, *n* (%)							
Vision loss	186 (100)	40 (100)	53 (100)	60 (100)	29 (100)	4 (100)	
Eye redness	83 (45)	11 (28)	26 (49)	27 (45)	19 (66)	0 (0)	**0.017**^c^
Eye pain	63 (34)	6 (15)	22 (42)	20 (33)	15 (2)	0 (0)	**0.008**^d^
Distorted vision	28 (15)	12 (30)	7 (13)	5 (8)	3 (10)	1 (20)	**0.042**^b^
Extraocular signs or symptoms, *n* (%)							
Auditory symptoms	40 (21)	2 (5)	11 (21)	16 (27)	11 (38)	0 (0)	**0.007**^c^
Neurological symptoms	121 (65)	24 (60)	41 (77)	35 (58)	19 (66)	2 (40)	0.194^b^
Integumentary finding	3 (2)	0 (0)	2 (4)	0 (0)	1 (3)	0 (0)	0.249^b^

*n*, number

^a^ one way ANOVA test

^b^ Fisher exact test

^c^ Chi-square test

### Onset-to-admission window

The mean interval between the symptom onset and the first visit to the hospital was 10.8 ± 9.2 days (range 1 – 90 days). The mean duration of corticosteroid treatment at the hospital was 8 days (range 3 – 21 days). The dosages of corticosteroids were adjusted and tapered gradually in the subsequent visits over a minimum of at least 9 months. The median follow-up period was 12 months (range 9 – 72 months).

### Chief complaints and disease manifestation at first visit

All admitted patients complained of blurred vision (100%), followed by red eye (45%), eye pain (34%), and distorted vision (15%). Among the extraocular findings, meningismus (65%) was the most common complaint and coincident with the onset of the ocular symptoms of the disease. Others included tinnitus (21%) and integumentary symptoms, such as alopecia, poliosis, and vitiligo (2%). The frequency of the chief complaint, such as the distorted vision, eye redness, eye pain, and auditory symptom category in Group 1, was significantly lower than those observed in other groups. All noted symptoms were presented in Table 1.

The most common anterior segment findings were anterior flare and anterior cells. For all admitted patients, 175 eyes (47%) showed no anterior chamber inflammation activity, and the rest showed mild anterior uveitis, with 140 eyes (38%) showing 1 + anterior chamber (AC) cell sign, 50 (13%) with 2 + , and 7 (2%) eyes with 3 + (Table 2). Most of the eyes within one-week of onset revealed negative AC sign (61% eyes for negative AC flare and cell). The AC activity increased as the onset time progressed, most eyes still presented mild activity with up to 1 + cell (Supplementary Fig. 2). Thirty-three eyes showed iris synechiae, and 90 eyes showed mutton fat keratic precipitates. The keratic precipitates sign was commonly observed in Group 4. For the posterior segment, most of the eyes showed mild vitritis, with 322 (87%) eyes showing 1 + vitreous reaction. Optic disc edema was observed in 314 (84%) eyes and exudative retinal detachment in 366 (98%) eyes. Only 2 eyes in Group 5 presented sunset glow fundus. The patients in Group 1 to Group 4 showed retinal manifestations, such as optic disc edema and retinal detachment (including serous retinal detachment and bacillary layer detachment), while the patients in Group 5 presented with less retinal detachment and more frequent sunset glow fundus (all adjusted* p* < 0.05). Table 2 summarized the slit-lamp and retinal manifestations.

**Table 2:** Disease manifestations at the first visit

	Total (372 eyes)	GROUP 1 (80 eyes)	GROUP 2 (106 eyes)	GROUP 3 (120 eyes)	GROUP 4 (58 eyes)	GROUP 5 (8 eyes)	*p-value*
Anterior segment, *n* (%)							
Anterior flare							** < 0.001**^a^
-	172 (46)	62 (77)	56 (53)	40 (33)	12 (21)	2 (25)	
+	141 (38)	15 (19)	36 (34)	54 (45)	30 (52)	6 (75)	
+ +	52 (14)	3 (4)	14 (13)	26 (22)	9 (15)	0 (0)	
+ + +	7 (2)	0 (0)	0 (0)	0 (0)	7 (12)	0 (0)	
Anterior cell							** < 0.001**^a^
-	175 (47)	61 (76)	52 (49)	46 (38)	14 (24)	2 (25)	
+	140 (38)	12 (15)	39 (37)	55 (46)	28 (48)	6 (75)	
+ +	50 (13)	7 (9)	13 (12)	19 (16)	11 (19)	0 (0)	
+ + +	7 (2)	0 (0)	2 (2)	0 (0)	5 (9)	0 (0)	
KP	90 (24)	12 (15)	(0)	35 (29)	26 (45)	0 (0)	** < 0.001**^b^
Iris nodules/synechiae	33 (9)	0 (0)	17 (16)	6 (5)	8 (14)	2 (25)	** < 0.001**^b^
Posterior segment, *n* (%)							
Vitreous opacity							** < 0.001**^a^
-	10 (2.7)	0 (0)	4 (4)	2 (2)	4 (7)	0 (0)	
+	322 (86.6)	78 (98)	92 (87)	108 (90)	38 (65)	6 (75)	
+ +	38 (10.2)	2 (3)	10 (9)	8 (6)	16 (28)	2 (25)	
+ + +	2 (0.5)	0 (0)	0 (0)	2 (2)	(0)	0 (0)	
Optic disc edema	314 (84)	60 (75)	88 (83)	106 (90)	54 (93)	6 (75)	0.083^b^
Retinal detachment	366 (98)	79 (99)	106 (100)	117 (99)	58 (100)	6 (75)	**0.003**^b^
Sunset glow fundus	2 (0.5)	0 (0)	0 (0)	0 (0)	0 (0)	2 (25)	** < 0.001**^b^
Dalen–Fuchs nodules	0 (0)	0 (0)	0 (0)	0 (0)	0 (0)	0 (0)	

*n*, number, *KP* keratic precipitates

^a^ Chi-square test

^b^ Fisher exact test

For the auxiliary examination shown in Table 3, FFA, OCT, B-scan ultrasonography, and UBM were collected if available. The OCT imaging revealed serous retinal detachment in almost all eyes (99.7%). RPE folds were also observed (72%). The median foveal thickness upon retinal detachment measured by OCT was 834 µm in 333 examined eyes though most (67%) were less than 200 µm (Table 4). The patients in Group 5 had significantly smaller detachment as compared to the other groups (all adjusted* p* < 0.05), which was consistent with the fundus manifestations. The FFA examination revealed early punctate staining and late subretinal dye pooling in most eyes, with 97% in Group 1, 98% in Group 2, 95% in Group 3, 100% in Group 4, and 50% in Group 5. The typical early punctate staining in FFA was primarily observed in the patients with one-month onset (adjusted* p* < 0.05 between Group 5 and all other groups). Optic disc hyperfluorescence was also common, varying from 76%—100% in different groups. The B-scan revealed retinal detachment in more than 80% of the studied eyes. For the UBM examination, the ciliochoroidal detachment (62% of 228 eyes) was the most common finding, followed by shallow anterior chamber (25%) and angle closure (18%), of which 8 (4.3%) eyes showed ocular hypertension with the range of 23 — 32 mmHg. No typical pattern in UBM was identified.

**Table 3:** Ancillary tests

		Total	GROUP 1	GROUP 2	GROUP 3	GROUP 4	GROUP 5	*p-value*
OCT, *n* (%) (335/67/94/110/56/8 eyes)	Serous retinal detachment	331 (99)	66 (99)	93 (99)	110 (100)	56 (100)	6 (75)	**0.002**^a^
	RPE folds	240 (72)	40 (60)	68 (72)	87 (79)	41 (73)	4 (50)	**0.044**^a^
FFA, *n* (%) (306/66/82/106/44/8 eyes)	Early punctate staining and late subretinal pooling	293 (96)	64 (97)	80 (98)	101 (95)	44 (100)	4 (50)	** < 0.001**^a^
	Optic disc hyperfluorescence	267 (87)	50 (76)	71 (87)	100 (94)	38 (86)	8 (100)	**0.009**^a^
B-scan ultrasonography, *n* (%) (250/46/78/80/40/6 eyes)	Retinal detachment	202 (83)	37 (80)	68 (87)	65 (81)	32(80)	6(100)	0.649^a^
UBM, *n* (%) (228/50/62/76/36/4 eyes)	Cells in AC	28 (12)	2 (4)	5 (8)	17 (22)	2 (6)	2 (50)	**0.002**^a^
	Closure of angle	42 (18)	6 (12)	11 (18)	17 (22)	8 (22)	0 (0)	0.559^a^
	Ciliary body edema	26 (11)	0 (0)	4 (6)	12 (16)	8 (22)	2 (50)	** < 0.001**^a^
	Ciliochoroidal detachment	142 (62)	30 (60)	32 (52)	50 (66)	26 (72)	4 (100)	0.129^a^
	Shallow AC	58 (25)	9 (18)	14 (23)	27 (35)	8 (22)	0 (0)	0.151^a^

*n* Number, *FFA* Fundus fluorescein angiography, *OCT* Optical coherence tomography, *UBM* Ultrasound biomicroscopy

^a^ Fisher exact test

**Table 4:** Foveal thickness on OCT

GROUP, *n* (%)	< 200um	200–500 um	500–1000 um	> 1000 um	Median detachment (IQR)
Total (333 eyes)	225 (67)	52 (15)	45 (13)	11 (3)	834 (279,929)
Group 1 (67 eyes)	46 (69)	7 (10)	12 (18)	2 (3)	754 (343,1166)
Group 2 (94 eyes)	59 (63)	16 (17)	16 (17)	3 (3)	766 (382,935)
Group 3 (110 eyes)	73 (66)	20 (18)	14 (13)	3 (3)	417 (250,835)
Group 4 (56 eyes)	43 (77)	8 (14)	2 (4)	3 (5)	685 (225, 675)
Group 5 (6 eyes)	4 (67)	1 (17)	1 (17)	0 (0)	150 (122, 934)

*n* number, *OCT* Optical coherence tomography

### Laboratory blood tests

Abnormal high white blood cell count was observed in 5 patients, among which 4 showed high neutrophils and 1 with high lymphocytes. Abnormal liver function with elevated alanine aminotransferase (ALT) and aspartate aminotransferase (AST) was observed in 3 patients, and elevation in the urea and creatinine levels was found in 3 patients. The patients received corticosteroid therapy under consultation with the internists. Fifteen of the 117 patients showed positive in the HLA-B27 test, and 15 of the 152 patients showed 1:100 positive in the ANA test. None of these patients complained of typical lupus symptoms, including unexplained rash, fatigue, or fever. Physical examination, including skin rash, arthritis, and renal dysfunction, was also negative. Diagnosis of lupus was ruled out in all enrolled patients. Further anti-ENA screening was conducted in 58 patients. Anti-Ro52 antibodies were detected in 6 patients, and anti-SS-A antibodies were detected in 2 patients. Anti-SS-B antibodies, anti-Scl70 antibodies, anti-chromatin antibodies, and anti-histone antibodies were respectively detected in 1 patient.

### Visual outcome and symptoms at the follow-up visits

The logMAR BCVA worse than 0.3 at presentation was found in 334 eyes (90%, Supplementary Fig. 3). At the 1-year follow-up visit, the logMAR BCVA of 0.1 or better was found in 111 of the 178 eyes (62%). The logMAR BCVA worse than 0.7 was found in 9 eyes (5%), and worse than 0.3 in 26 eyes (15%) at 1 year after the onset. The logMAR BCVA was significantly improved from 0.74 ± 0.54 at baseline to 0.12 ± 0.24 at the 1-year follow-up visit (*p* < 0.001, Table 5). Significant better BCVA at the 1-year follow-up visit was observed in all groups, except Group 4 (all *p* < 0.001 for Group 1, 2, and 3; Group 5 was unavailable due to the loss of the follow-up visits). Two patients complained of vitiligo over the follow-up visits.

**Table 5:** BCVA at the baseline and the one-year follow-up

GROUP	Baseline BCVA	One-year BCVA	*P value*
Total (*n* = 178)	0.74 ± 0.54	0.12 ± 0.24	** < 0.001**
Group 1 (*n* = 80)	0.72 ± 0.46	0.10 ± 0.21	** < 0.001**
Group 2 (*n* = 8)	0.72 ± 0.62	0.11 ± 0.29	** < 0.001**
Group 3 (*n* = 36)	0.75 ± 0.59	0.13 ± 0.16	** < 0.001**
Group 4 (*n* = 4)	1.06 ± 0.41	0.42 ± 0.47	0.087

*n* number, *BCVA* Best corrected visual acuity

### Recurrence of VKH

During the follow-up period for at least 9 months, the recurrence of inflammation was observed in 34 (18%) out of 186 patients, including 4 patients (10%) in Group 1, 7 patients (13%) in Group 2, 9 patients (15%) in Group 3, 12 patients (41%) in Group 4, and 2 patients (60%) in Group 5. The association analyses on the recurrences with different laboratory parameters identified high ESR (*p* = 0.017) and C-reactive protein (*p* = 0.020) significantly correlated with VKH recurrences (Table 6).

**Table 6:** Recurrence and laboratory tests

	ASO( +)	ASO(-)	RF ( +)	RF (-)	ESR ( +)	ESR (-)	CRP ( +)	CRP (-)	HLA B27( +)	HLA B27(-)	ANA ( +)	ANA (-)	ENA ( +)	ENA (-)
Recurrence ( +)	0	31	1	17	10	21	5	21	4	17	3	26	2	10
Recurrence (-)	9	113	5	76	17	105	4	93	11	84	12	110	10	35
*P*	0.205^a^		1^a^		**0.017**^b^		**0.02**^**a**^		0.470^a^		1^a^		1^a^	

*ESR* erythrocyte sedimentation rate, *ASO* Anti-streptolysin O titer, *HLA-B27* Human leukocyte antigen B27, *ANA* Antinuclear antibody, *ENA* Extractable nuclear antigen

^a^ Fisher exact test

^b^ Chi-square test

## Discussion

This retrospective study investigated the patients with VKH disease in the acute phase admitted to our eye hospital in southern China. The onset age of the patients was around 40 years old, similar to other studies [1, 7]. The ratio of male to female was similar to that in another study on southern Chinese subjects [7]. Equal male and female distribution was also reported in Japan and Singapore [4, 18]. Most enrolled patients were diagnosed with incomplete VKH, and it is consistent in most studies using the revised diagnostic criteria [1]. If the patients can be diagnosed and treated in the early stage, they are less likely to develop the integumentary signs and thus complete VKH. The integumentary signs were rare in this study, but it is relatively reasonable considering the short interval of the symptom onset and the initial evaluation.

**Fig. 1:**
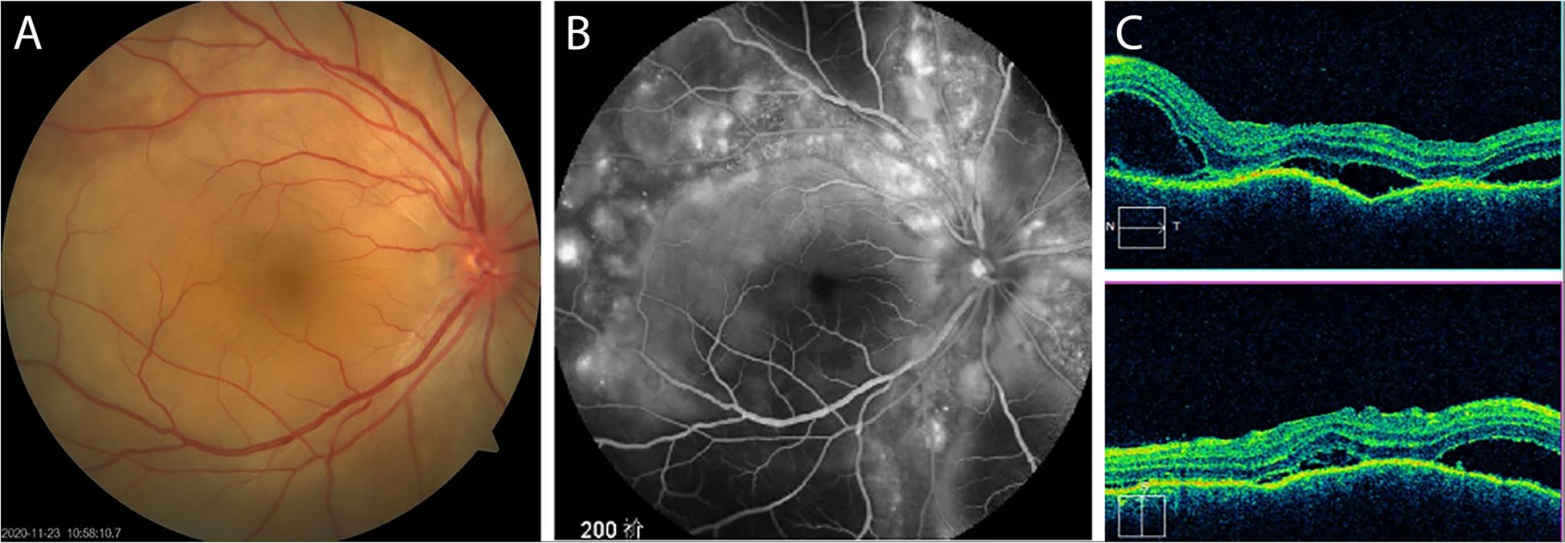
Clinical findings in the acute phase of VKH disease. **A**. Fundus photo shows the optic disc hyperemia, white-yellowish choroidal lesions, and localized exudative retinal detachments. **B**. FFA shows optic disc hyperfluorescence and the subretinal pooling of the dye in the late phase. **C**. OCT shows exudative retinal detachment. FFA, fundus fluorescein angiography; OCT, optical coherence tomography

Most of the studies on the VKH disease were carried out in referral centers or tertiary hospitals, where the patients were referred after the symptom onset [4, 7, 19]. The present study was carried out in a regional eye hospital. The enrolled patients visited the hospital within the early onset of uveitis (82% within 2 weeks); thus, we were able to explore the potential changing pattern of the disease at the early stage. We observed that the anterior segment was not involved at the very early stage (within 4 days), and the anterior chamber activity increased slightly as the onset time prolonged, with mild anterior uveitis in most cases. For the posterior segment, almost all eyes in the acute phase presented with serous retinal detachment with or without optic disc edema. The patients within the onset of one month presented with more common retinal detachment and more extensive fovea detachment. Therefore, we postulated that the patients with the VKH disease first presented with typical serous retinal detachment followed by anterior chamber activity. Similarly, our patients showed an earlier anterior segment involvement as compared to those with onset within 2 weeks in a previous study [7]. The ancillary tests supported the clinical diagnosis of the VKH disease. Consistent with other studies, the FFA examination showed the classical early punctate staining and late subretinal dye pooling, whereas the B-scan and OCT imaging revealed serous retinal detachment (Fig. 1) [5, 7, 18].

The laboratory tests can assist in the underlying pathological diagnosis and identifying the potential systemic diseases [20]. In the present study, the visual outcome of the patients did not differ dramatically between the patients with and without the presence of inflammatory or immunological markers. Further studies are needed to confirm the potential association as the patients were not routinely tested with various laboratory tests.

Recurrence rates vary in different studies [21]. A previous study in China reported a 15% recurrence rate using a high dose of corticosteroid mostly combined with other immunosuppressive agents for about 1 year, and recurrences drop using a novel reduced dose of corticosteroids combined with immunosuppressive therapy in Chinese patients [22]. About 25% of Japanese VKH patients receiving high dose corticosteroid therapy suffer recurrent inflammation [8]. In this study, the patients in the acute phase were treated solely with oral corticosteroid tapered within 9 months, and 18% of the patients showed recurrence during and after the treatment. Therefore, we postulated that early treatment could reduce recurrences with the application of oral corticosteroid therapy. Recurrence of uveitis might be related to the initial visual acuity, the window time to the first treatment, and different treatment regimens [21]. Long-term studies are needed to determine the best treatment regimens considering both final visual outcome and recurrence.

The outcome of BCVA at the 1-year follow-up visit was promising for most patients who received oral corticosteroid therapy. We believed that the visual prognosis of VKH is favorable if early appropriate therapy can be initiated [14, 23]. In a Japanese study on acute VKH patients, 93% of the eyes treated with pulse intravenous corticosteroids achieved a visual acuity of ≥ 1.0 at 1 year after presentation [18]. Although the routes and doses of corticosteroid administration remain to be investigated, prompt and aggressive corticosteroid treatment with slow tapering over is generally recommended [1].

In summary, this study presented the clinical characteristics and visual outcomes of patients with the VKH disease in the acute phase. Most of the patients visited the hospital with a complaint of blurred vision. At the onset within 1 week, anterior uveitis is not common. As the disease progresses, anterior segment can be involved in most cases. Moreover, multifocal serous retinal detachment can always be observed within the onset of one month. The possibility of VKH should be noted when the above manifestations are detected at the clinic. The ancillary tests, such as OCT and FFA, are crucial to confirm the diagnosis at an early stage. Visual prognosis is favorable if early and aggressive treatment can be given.

## Supplementary Information

**Additional file 1: Supplementary** **Fig. 1. **Age and gender distribution of the patients with acute VKH disease at diagnosis.

**Additional file 2: Supplementary** **Fig. 2.** Anterior chamber activity of the patients with acute VKH disease in different groups.

**Additional file 3: Supplementary Fig. 3.** BCVA at presentation and one-year follow-up in patients with acute VKH disease. BCVA, best corrected visual acuity.

## Data Availability

The datasets used and/or analysed during the current study are available from the corresponding author on reasonable request.

## Abbreviations

VKH: Vogt-Koyanagi Harada
BCVA: Best corrected visual acuity
IOP: Intraocular pressure
FFA: Fundus fluorescein angiography
OCT: Optical coherence tomography
UBM: Ultrasound biomicroscopy
CBC: Complete blood count
ESR: Erythrocyte sedimentation rate
ASO: Anti-streptolysin O titer
HLA-B27: Human leukocyte antigen B27
ANA: Antinuclear antibody
ENA: Extractable nuclear antigen
AC: Anterior chamber
ALT: Alanine aminotransferase
AST: Aspartate aminotransferase
logMAR: Logarithm of the minimum angle of resolution
