# Supplementary figures and images for: Acute phase clinical manifestations of patients with Vogt-Koyanagi-Harada disease in Southern China
Source: BMC Ophthalmol. 2023 May 5;23:199. doi: 10.1186/s12886-023-02952-y (PMC10161656; doi:10.1186/s12886-023-02952-y)

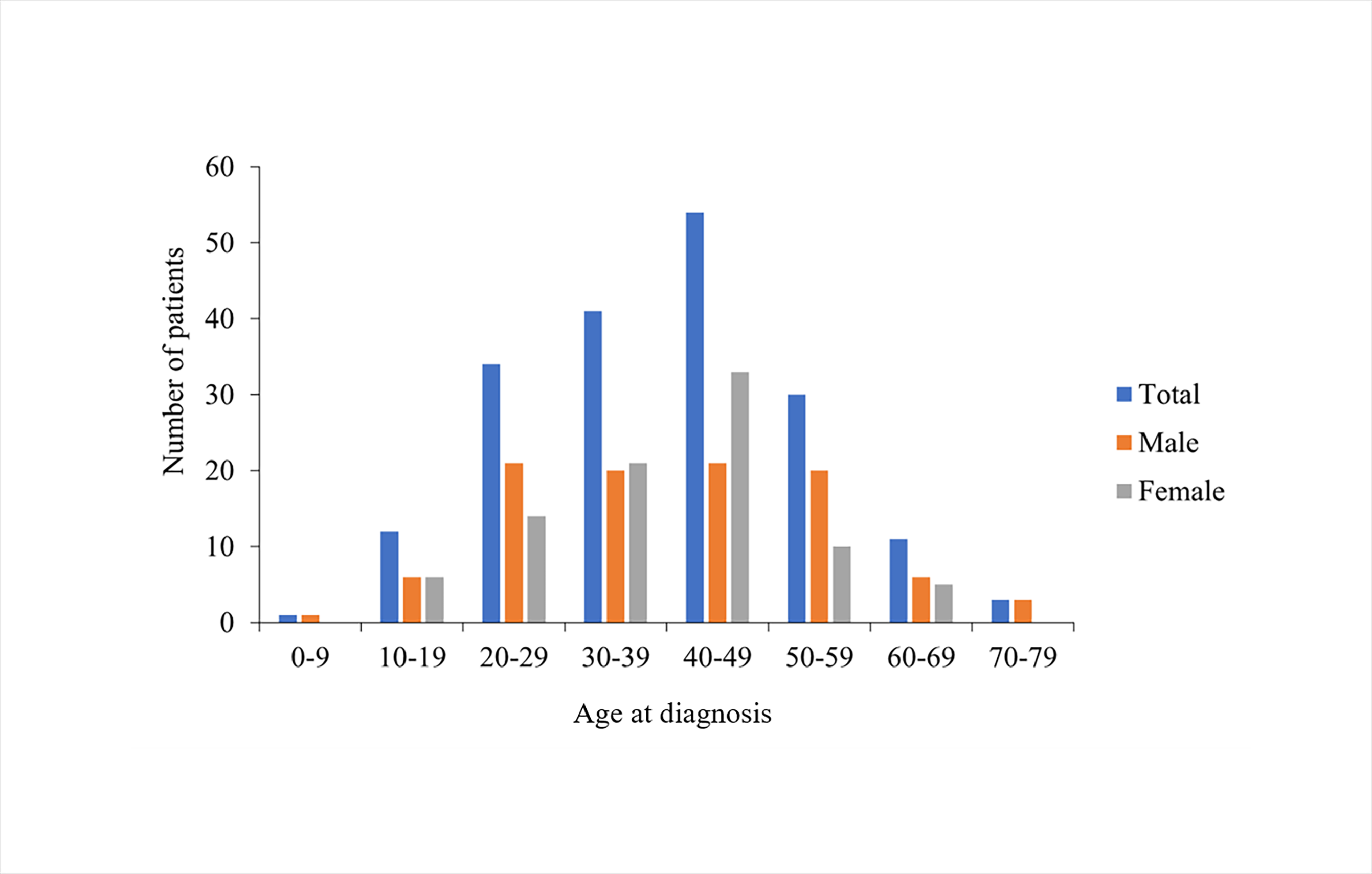

Supplement: Supplementary file 1 — Additional file 1: Supplementary Fig. 1. Age and gender distribution of the patients with acute VKH disease at diagnosis. [file 12886_2023_2952_MOESM1_ESM.tif]

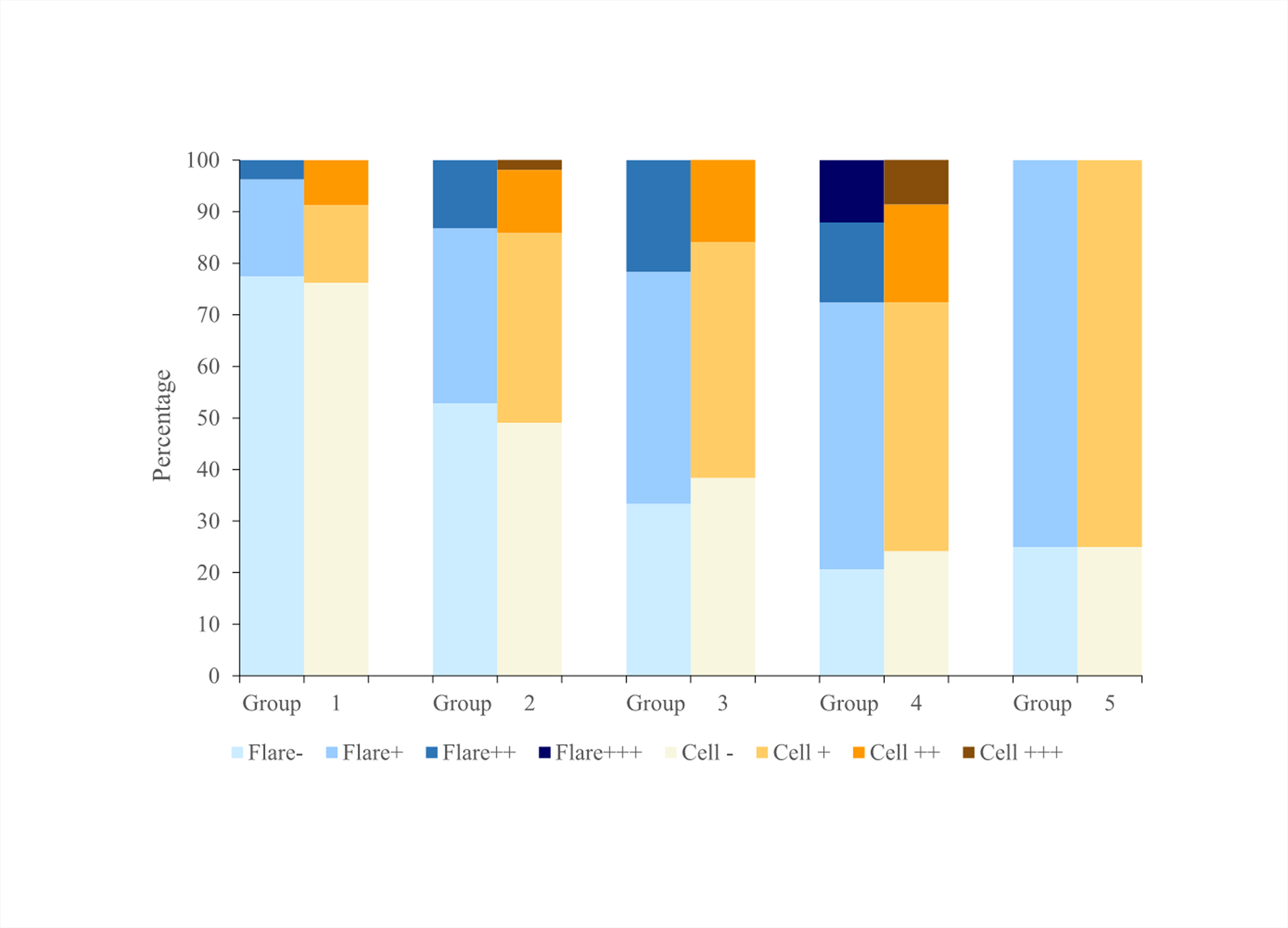

Supplement: Supplementary file 2 — Additional file 2: Supplementary Fig. 2. Anterior chamber activity of the patients with acute VKH disease in different groups. [file 12886_2023_2952_MOESM2_ESM.tif]

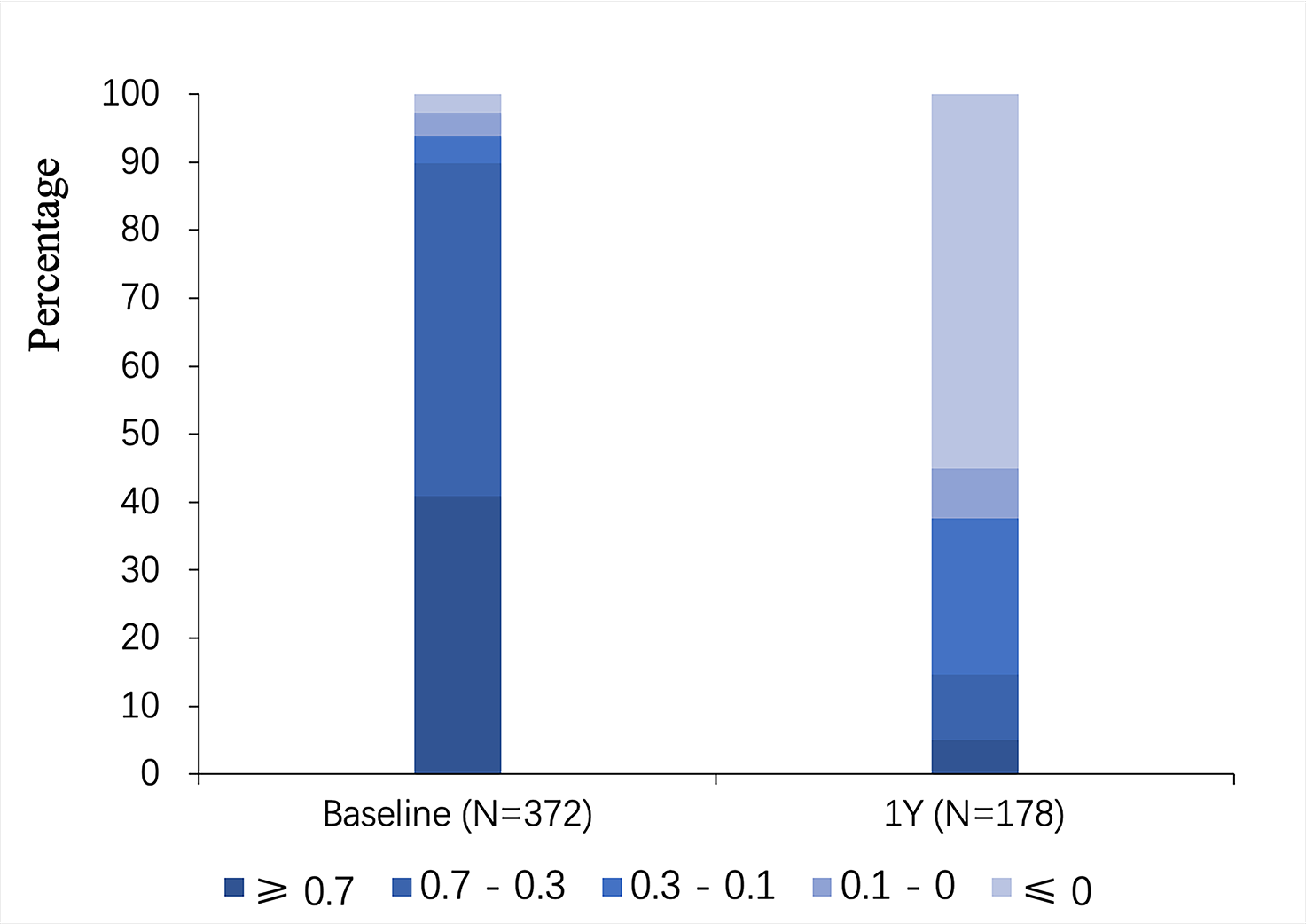

Supplement: Supplementary file 3 — Additional file 3: Supplementary Fig. 3. BCVA at presentation and one-year follow-up in patients with acute VKH disease. BCVA, best corrected visual acuity. [file 12886_2023_2952_MOESM3_ESM.tif]
